# Genomic insights into the resistome, mobilome and functional adaptation of Achromobacter xylosoxidans across clinical and environmental contexts

**DOI:** 10.1099/mgen.0.001744

**Published:** 2026-06-29

**Keywords:** *Achromobacter xylosoxidans*, antibiotic resistance, genomics, horizontal gene transfer, opportunistic pathogen

## Abstract

*Achromobacter xylosoxidans* is an emerging opportunistic pathogen associated with a wide range of infections in humans. This species is widely distributed in the environment due to its high adaptability. Isolates of *A. xylosoxidans* have intrinsic resistance to several antibiotics and the potential to acquire genetic resistance determinants. Despite its growing frequency of isolation, little is known about the genomic characteristics of this pathogen. In this study, we conducted a comprehensive genomic analysis of assemblies from the NCBI RefSeq database, along with a newly sequenced respiratory isolate from a patient with cystic fibrosis. Through pangenome analysis, we identified genes and functions associated with specific isolation sources, suggesting niche-specific adaptation. Resistance-associated mutations in the AxyZ efflux pump regulator, along with *bla*_AXC-1_, were exclusively detected in genomes of clinical origin. Furthermore, while the resistome is limited, non-core antimicrobial resistance genes were detected to be primarily associated with the mobilome, underscoring the potential for horizontal gene transfer to further shape resistance in this species.

**Impact Statement**

The frequency of infections associated with *Achromobacter xylosoxidans* has increased in recent years, particularly in people with cystic fibrosis. Despite its growing clinical importance, the genomic basis of its antibiotic resistance and adaptability remains poorly understood, as large-scale comparative genomic analyses of this genus are still scarce. In this study, we performed comparative genomics of publicly available *A. xylosoxidans* genomes and a newly sequenced clinical isolate to investigate the genetic basis of antibiotic resistance and environmental adaptability. Mutations in the *axyZ* efflux pump regulator, along with *bla*_AXC-1_, were exclusively detected in clinical isolates, independent of their lineage. These mutations are likely associated with antimicrobial resistance phenotypes. Although the core resistome is conserved, diversity in accessory resistance genes is driven by the mobilome. These findings provide a genomic framework for understanding the spread and evolution of *A. xylosoxidans*.

## Data Summary

The raw sequencing reads and the genome assembly of the sequenced *Achromobacter xylosoxidans* are available in the NCBI repository with the BioSample ID SAMN50443078. The authors confirm that all supporting data, code and protocols have been provided within the article or through supplementary data files.

## Introduction

*Achromobacter xylosoxidans* is a Gram-negative bacterium capable of persisting in diverse environments, including hospital settings, despite conventional disinfection protocols [1]. It is an opportunistic pathogen that primarily affects immunocompromised individuals with chronic respiratory diseases, with colonization rates of ~10% in patients with cystic fibrosis (CF) [2,4].

Clinical isolates of *A. xylosoxidans* may form biofilms and exhibit intrinsic antimicrobial resistance (AMR) to several antibiotics [5,6]. This AMR has been associated with the resistance–nodulation–division-type efflux system AxyXY-OprZ [7,8]. Overexpression of this efflux pump has been associated with loss-of-function mutations in its negative regulator *axyZ*, which promotes antibiotic expulsion [7,9].

The adaptability of this organism to non-suitable environmental conditions has allowed the acquisition of foreign AMR, facilitated by mechanisms of horizontal gene transfer (HGT) [10] such as mobile genetic elements (MGEs), integrons, plasmids, and prophages [11]. All these factors are thought to contribute to its genomic plasticity [12].

Most genomic studies of *A. xylosoxidans* have focused on the longitudinal characterization of strains, particularly those isolated from CF patients, or on specific aspects of its biology, leaving significant gaps in the understanding of its genetic repertoire and evolutionary dynamics. Despite the growing clinical significance of *A. xylosoxidans* and documented genomic complexity, the understanding of the genetic basis of its AMR, pathogenicity, virulence mechanisms and adaptive capabilities remains incomplete [11,13, 14].

In this study, we conducted a comparative virulome, resistome, mobilome, pangenomeand phylogenetic analysis of *A. xylosoxidans* by using publicly available assemblies and a newly sequenced CF clinical isolate.

## Methods

### Whole-genome sequencing and assembly

One cryopreserved *A. xylosoxidans* respiratory isolate, collected in 2012 from a paediatric CF patient, was cultured on MacConkey agar. Genomic DNA was extracted using the phenol–chloroform method. Whole-genome sequencing was performed on an Illumina NovaSeq 6000 platform by an external service (Novogene, CA, USA). Read quality was verified using FASTQC v0.12.1 [15], quality-filtered with Trim_Galore v0.6.10 [16] and assembled using Unicycler v0.5.1 [17]. Assembly quality was verified using QUAST v5.2.0 [18], and genome completeness and contamination were assessed using CheckM v1.2.3 [19]. The assembly is available in the NCBI under the SAMN50443078 BioSample identifier.

### Public assembly retrieval, taxonomy and functional annotation

Publicly available *A. xylosoxidans* genomes, along with their corresponding protein sequences, were retrieved from the NCBI using the datasets v16.22.0 command-line tool. RefSeq assemblies were preferred over GenBank assemblies because the RefSeq database provides higher levels of curation and reduced redundancy (https://www.ncbi.nlm.nih.gov/refseq/about/). Duplicated genomes from longitudinal studies were removed, retaining only the earliest isolate in each case. Assembly quality was verified using QUAST v5.3.0 and CheckM v1.2.3 [19] for completeness and contamination.

Taxonomy was verified by calculating average nucleotide identity (ANI) between the retrieved assemblies and reference assemblies of *Achromobacter* species using ANIclustermap v2.0.1 [20]. ANI values greater than 95% were considered adequate for species assignment [21]. For ANI values lower than 95%, taxonomy was investigated using TYGS (https://tygs.dsmz.de/). Furthermore, core gene alignments of reference genomes and the included assemblies were obtained using Panaroo v1.5.1 [22] and subjected to maximum-likelihood (ML) phylogenetic analysis with IQ-TREE2 v2.3.6 [23] and visualized using FigTree (http://tree.bio.ed.ac.uk/software/figtree/). The best-fit substitution model was determined for each gene within the alignment with the Model Finder module, and branch support was estimated by 1,000 ultrafast bootstrap replicates. Functional annotation of protein sequences was performed with EggNOG-mapper [24] to analyse Clusters of Orthologous Genes (COG) and KEGG Orthology (KO) terms using the ClusterProfiler package [25].

### Multilocus sequence typing, pangenome and phylogenomic analyses

Multilocus sequence typing (MLST) was performed with the MLST command-line tool [26], which uses PubMLST databases. Assemblies were annotated using Prokka [27] and used to construct a pangenome and a core-gene alignment using Panaroo v1.5.1 [22] using a core gene threshold of ≥98%. IQ-TREE2 v2.3.6 [23] was used to construct an ML tree using the method described in the previous section. Additionally, assemblies were mapped to the *A. xylosoxidans* FDAARGOS_1091 genome (GCF_016728825.1) using Snippy v4.6.0 [28] to obtain a whole-genome SNP alignment. A core-SNP alignment was derived by masking recombination sites of the whole-genome alignment with Gubbins v3.2.1 [29] and extracting SNPs with SNP-sites v2.5.1 [30]. Finally, an SNP-distance matrix was constructed using SNP-dists v0.8.2 [31].

### Resistome, virulome and mobilome

AMR genes were identified using AMRFinderPlus v4.0.23 [32], the Resistance Gene Identifier v6.0.3 [33] and ABRicate v1.0.1. For ABRicate [34], the built-in Comprehensive Antibiotic Resistance Database (CARD) [33] and MEGARes [35] databases were employed. Output reports from each tool were parsed and merged using hAMRonization 1.1.9 [36]. To analyse specific AMR determinants, the corresponding protein sequences were identified in the annotated proteomes using blastp v2.12.0 [37], extracted using the SeqKit v0.8.1 grep function [38] and aligned with MAFFT v7.525 [39] for comparative analysis. Virulence factor (VF) genes were identified using ABRicate, using sequences from the Virulence Factor Database (VFDB) [40]; coverage values >80% were considered positive.

MGEs, including transposon, insertion sequence (IS), integrative conjugative element and integrative mobilizable element, were identified using Mobile Element Finder v1.1.2 [41]. Prophages and phage-like regions were detected with the PHASTEST docker image [42]. Plasmid sequences were reconstructed from assemblies using MOB-Suite v3.1.9 [43], and integrons were identified with Integron Finder v2.0.6 [44]. Genomic islands (GIs) were predicted using the IslandViewer 4 API [45]. Mobilome sequences were further screened for AMR and VF genes using the previously described approach. For comparative analysis of related mobilome sequences, mash distances were estimated with Sourmash v4.8.9 [46], and clusters with a similarity >0.9 for MGEs and >0.4 for phage sequences were extracted. To quantify the association between AMR genes and the mobilome, all MGE sequences per genome were concatenated and annotated using Prokka.

The proportion of MGE-associated genes was compared between AMR and non-AMR genes across all genomes using Fisher’s exact test in RStudio v4.4.2 [47]. Coding sequences (CDSs) from the whole genome were obtained from the Prokka annotation, while mobilome CDSs were obtained by concatenating all mobilome sequences per genome, annotating them with Prokka and dereplicating the resulting CDS using CD-HIT-EST v4.8.1 [48] to account for redundancy arising from nested mobile elements.

## Results

### Taxonomy verification and dataset composition

**Fig. 1.**
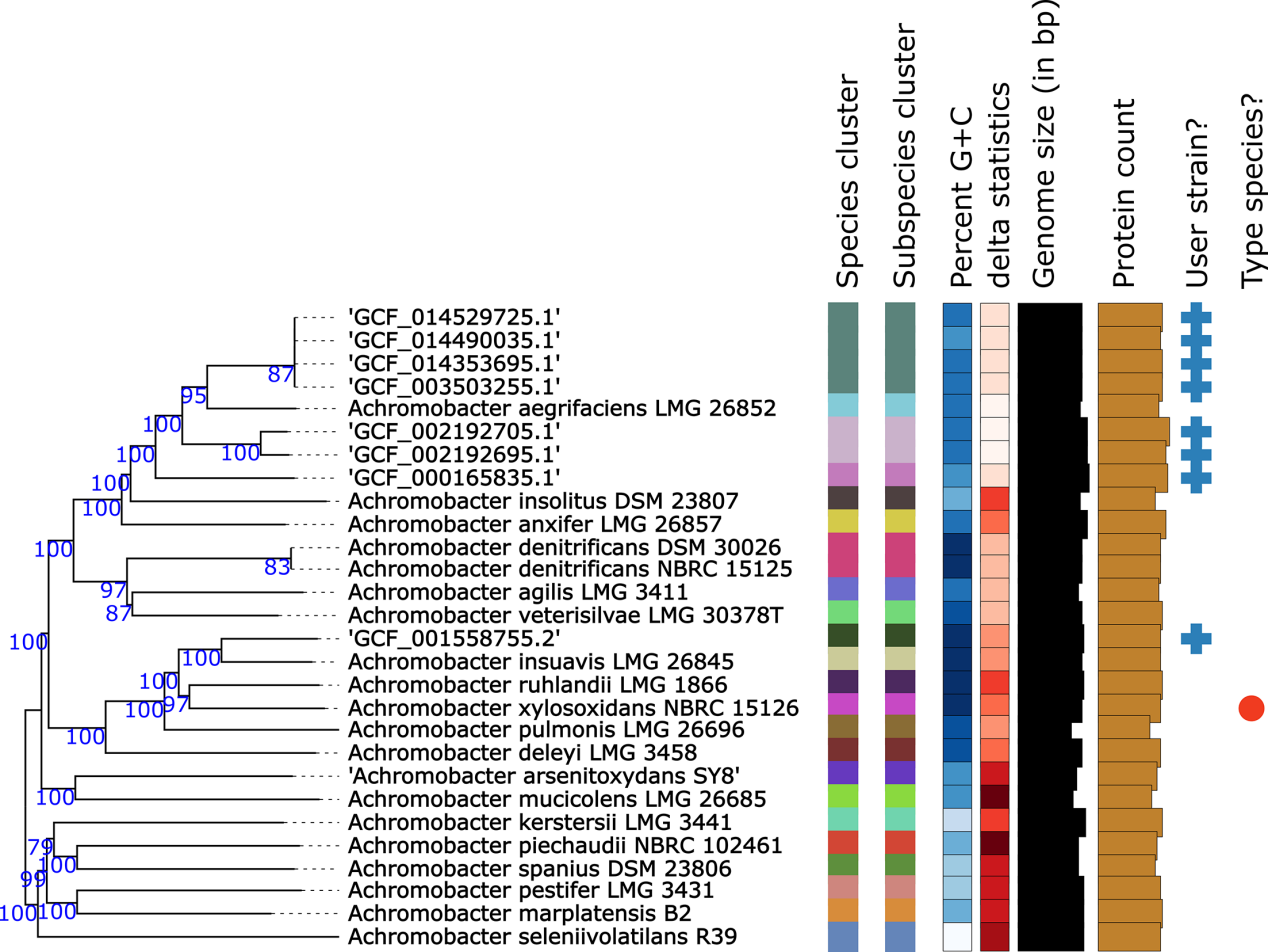
Results from the Type Strain Genome Server for assemblies with inconclusive taxonomy. Seven assemblies retrieved from the RefSeq database represent four putative novel species.

The initial dataset consisted of 154 assemblies, including the newly sequenced isolate. The taxonomy was verified by calculating the ANI of these genomes along with 22 type strains from different *Achromobacter* species (Fig. S1, available in the online Supplementary Material). Seven genomes had ANI below 95% with all type strains included (Fig. S2). These assemblies were uploaded to the TYGS, and results suggest that these strains represent novel species (Fig. 1). These seven strains formed four clades, all of which are closely related to *Achromobacter aegrifaciens* and *Achromobacter insolitus*. The first clade is formed by four genomes (GCF_014259725.1, GCF_014490035.1, GCF_01435695.1 and GCF_003503255.1), the second by two genomes (GCF_002192705.1 and GCF_002192695.1) and two separate clades with a sole representative each (GCF_000165835.1 and GCF_001558755.2). For this reason, these seven genomes were excluded from the final dataset.

After filtering non-redundant genomes, 115 assemblies with ANI values >95% were included for further analysis. Metadata for the analysed dataset is shown in Table S1. Assemblies were categorized by isolation source, with some of them being clinical (*n*=100), including CF respiratory (*n*=71), non-CF respiratory (*n*=10) and clinical non-respiratory (*n*=19). Environmental isolates were also included (*n*=12), and three isolates had missing data.

### Genomic features

An average completeness of 98% and contamination of 1.4% were obtained. Excluding complete genomes, the average number of contigs was 174, with an average L50 of 27 and N50 of 443,573 bp (Table S1). The average genome size was 6.6 Mb, with an average of 5,948 genes (± 215 genes) and a 67% GC content. The pangenome comprised 15,594 genes, of which 4,257 were core, 653 were soft core, 1,665 were shell and 9,019 were cloud genes. The core genome alignment spans ~4.2 Mb, representing ~63% of the genome length.

**Fig. 2.**
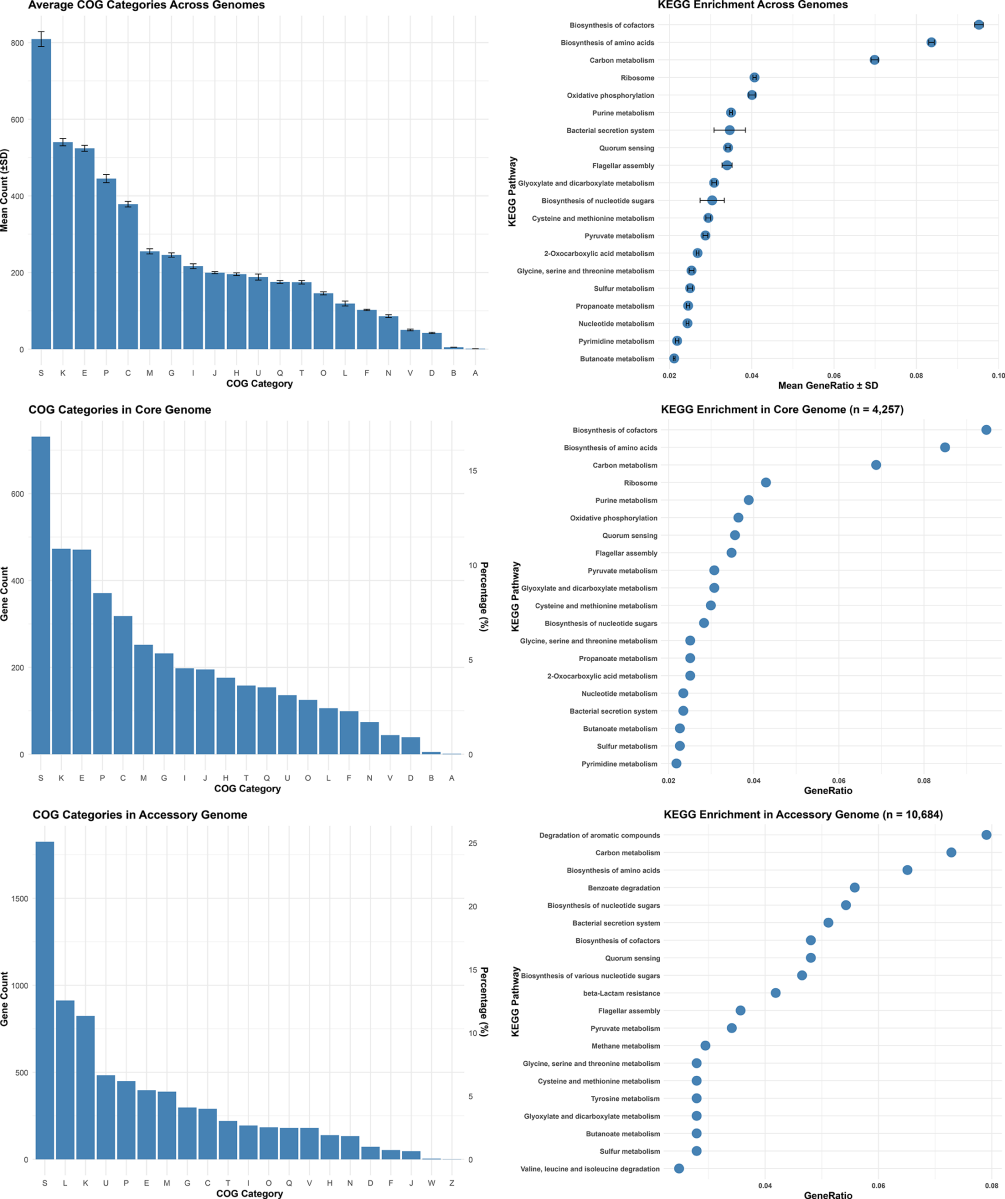
COG annotations and KEGG pathway enrichment analysis. The analysis was performed using the full set of annotated genomes, restricted to the core genome and the accessory genome (A: RNA processing and modification; B: chromatin structure and dynamics; C: energy production and conversion; D: cell cycle control, cell division, chromosome partitioning; E: amino acid transport and metabolism; F: nucleotide transport and metabolism; G: carbohydrate transport and metabolism; H: coenzyme transport and metabolism; I: lipid transport and metabolism; J: translation, ribosomal structure and biogenesis; K: transcription; L: replication, recombination and repair; M: cell wall/membrane/envelope biogenesis; N: cell motility; O: posttranslational modification, protein turnover and chaperones; P: inorganic ion transport and metabolism; Q: secondary metabolites biosynthesis, transport and catabolism; R: general function prediction only; S: function unknown; T: signal transduction mechanisms; U: intracellular trafficking, secretion and vesicular transport; V: Defence mechanisms; W: extracellular structures; Y: nuclear structure; Z: cytoskeleton).

Functional annotations of all genomes showed that the most abundant COG categories were of unknown function, transcription, amino acid metabolism, inorganic ion transport and energy production (Fig. 2). For KEGG pathway analysis, biosynthesis of cofactors, amino acids and carbon metabolism were the most enriched pathways across all genomes, with low sd and gene ratios greater than 0.07. The highest variability was observed for the bacterial secretion system (min=0.023, max=0.041) and biosynthesis of nucleotide sugars (min=0.025, max=0.035).

Comparison between the core and accessory genome annotations revealed that the core genome mirrors the average results from the full dataset, both for the COG annotations and the KEGG pathway analysis. The accessory genome was enriched in additional categories related to replication, recombination and repair; intracellular trafficking, secretion and vesicular transport; cell wall/membrane/envelope biogenesis; and carbohydrate metabolism. For KEGG pathway analysis, the accessory genome was enriched in pathways related to degradation of aromatic compounds, carbon metabolism, benzoate degradation, biosynthesis of nucleotide sugars, bacterial secretion system and quorum sensing (Fig. 2).

**Fig. 3.**
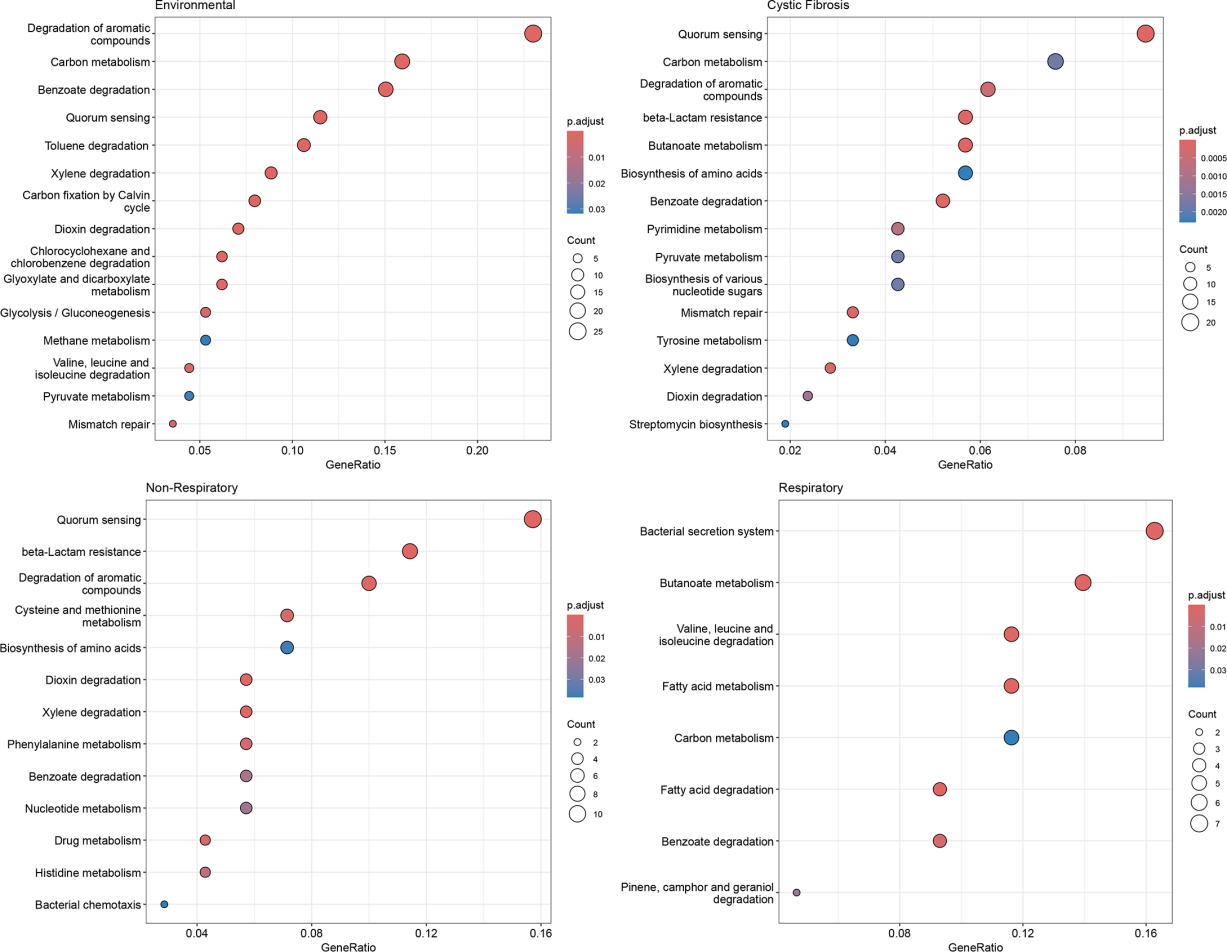
KEGG pathway enrichment analysis of all genes unique to genomes according to their isolation source.

KEGG pathway enrichment analysis, performed on the full gene set from each isolation source group, revealed distinct functional profiles (Fig. 3). Benzoate degradation was enriched across all groups, while quorum sensing and degradation of xylene, dioxin and aromatic compounds were enriched in all groups except the non-CF respiratory isolates. Unique pathway enrichments were observed for each group: non-CF respiratory isolates were enriched in pathways for secretion systems and fatty acid metabolism; clinical non-respiratory isolates showed enrichment in drug metabolism and chemotaxis; biosynthesis of nucleotide sugars was unique to CF isolates; and environmental isolates were enriched in the degradation of toluene, chlorocyclohexane and methane. Additionally, each group displayed enrichment in multiple pathways associated with amino acid and carbohydrate metabolism.

To focus on core functional content, the analysis was restricted to genes present in at least 95% of the genomes within each group. This analysis revealed 10 genes unique to non-CF respiratory isolates, including four hypothetical proteins. Three genes were involved in iron uptake (*fpvA*, *fecR* and *fecI*), while the remaining genes (*gabR*, *azoR* and *ephA*) were involved in gamma-aminobutyric acid metabolism, thiol-induced stress resistance and epoxide detoxification, respectively (https://www.uniprot.org/). The *wspC* gene, involved in biofilm formation, was the only gene unique to respiratory CF isolates.

### MLST and phylogenetic analysis

The most frequent sequence type (ST) was ST182 (*n*=10), followed by ST20 (*n*=6); ST175 and ST290 were represented by four assemblies each and three assemblies for ST346, ST327, ST314 and ST2. Most of the dataset was represented by STs of unique occurrences (*n*=28) and novel STs (*n*=39).

**Fig. 4.**
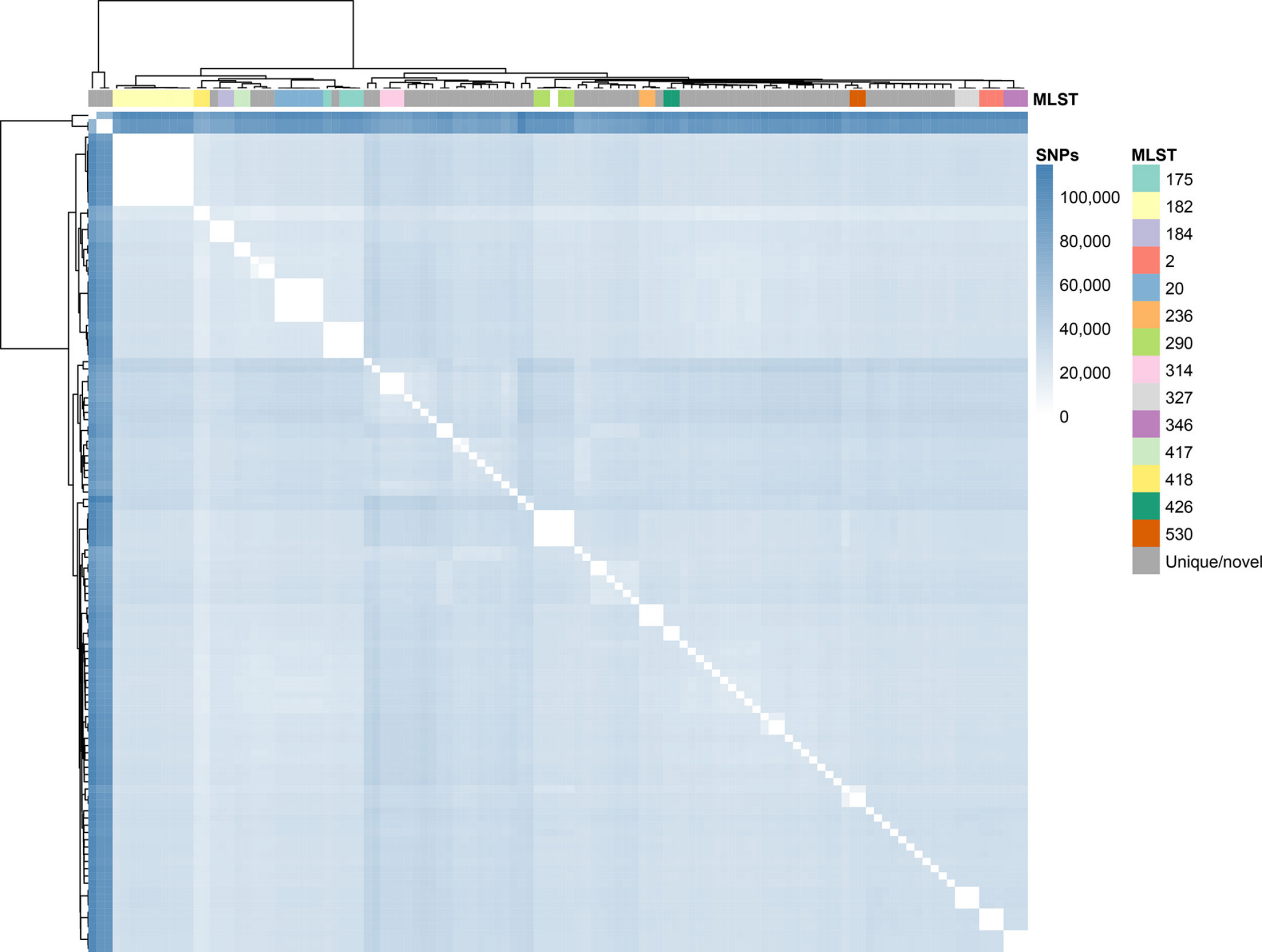
Recombination-masked SNP distance matrix. Colours depict clusters of the same ST; unique and novel STs are represented in grey for simplicity.

An average distance of 32,012 SNPs was observed between isolates, with the highest value at 114,460 SNPs and the lowest at 1 SNP. Clusters of the same STs were analysed to define the average intra-cluster SNPs (Fig. 4), for which STs with a frequency higher than three were considered. ST182 had an average of 124 SNPs (maximum=212, minimum=40), ST20 had 19 SNPs (maximum=55, minimum=1), ST175 had 86 SNPs (maximum=121, minimum=51) and ST290 had an average of 91 SNPs (maximum=107, minimum=64). Considering all STs with two or more genome representatives, the average intra-ST SNP-distance was 142 SNPs.

**Fig. 5.**
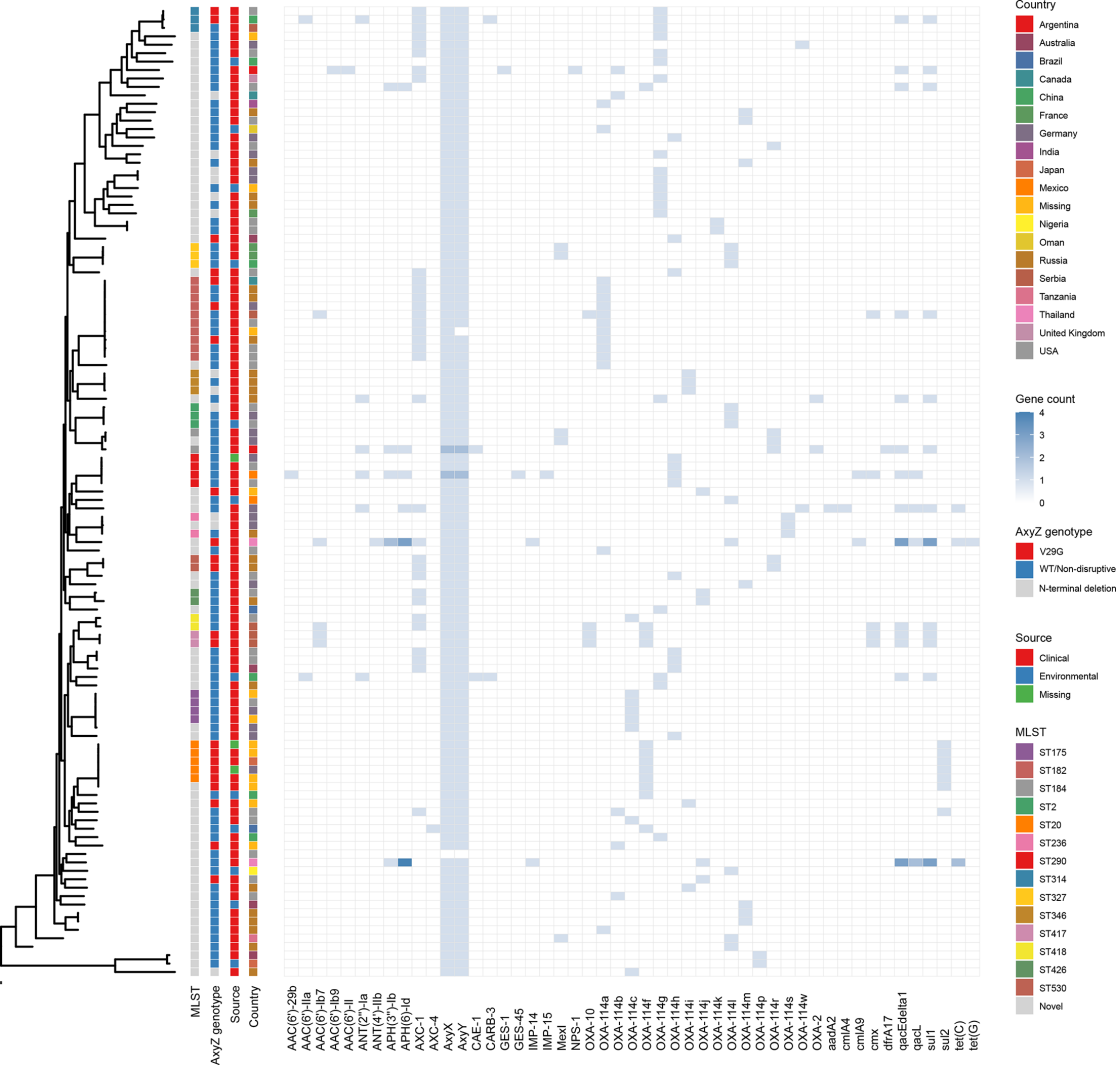
Midpoint-rooted ML phylogenetic tree based on the core genome alignment and AMR gene content.

The core-genome phylogenetic analysis shows the formation of clades of near-0 distance represented by the same STs (Fig. 5). In all cases, STs were not limited to a single geographic origin.

### Resistome and virulome

**Fig. 6.**
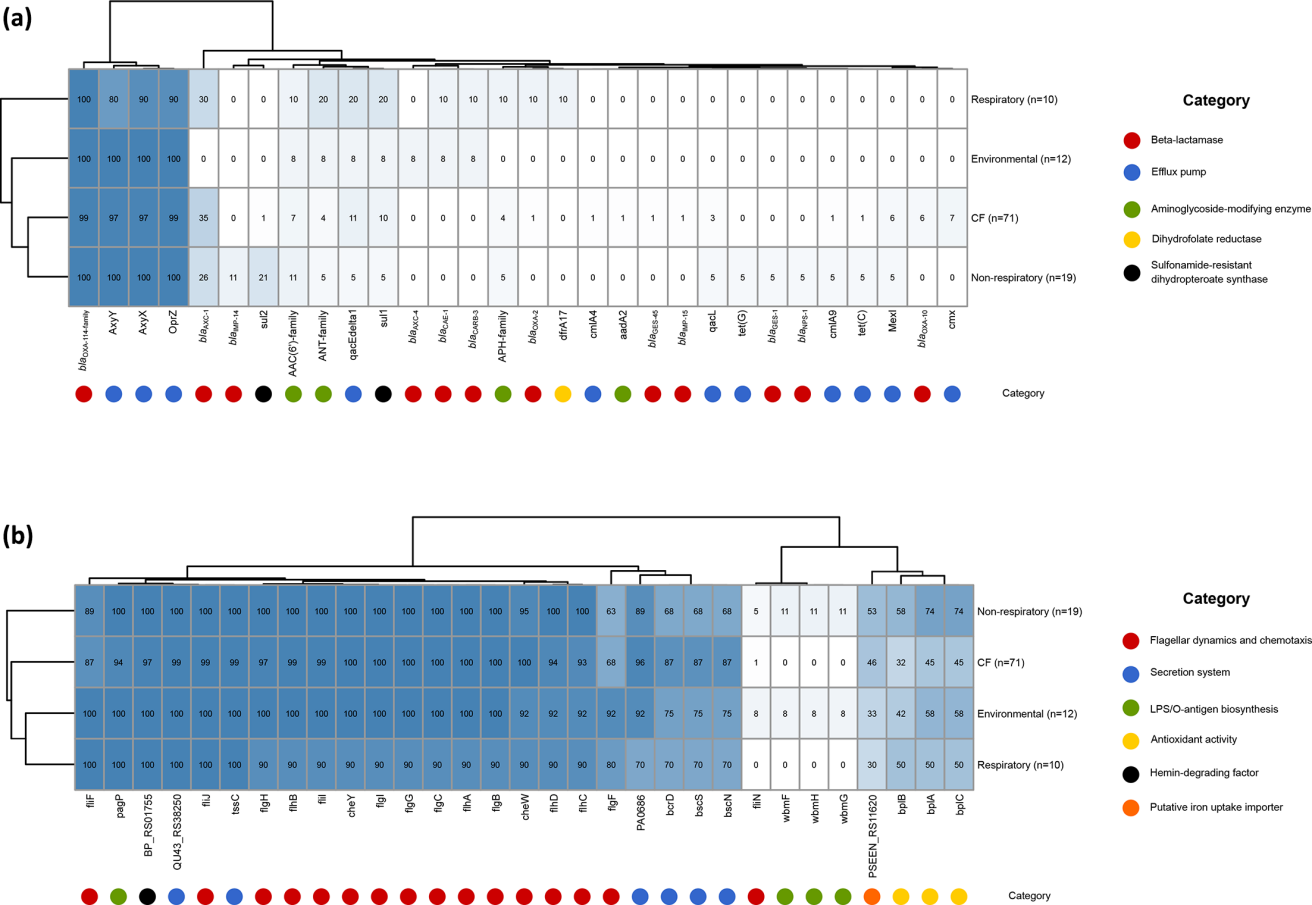
Resistome and virulome genes across isolation source groups. Values inside the squares stand for the relative frequency of the corresponding gene of the (a) resistome and (b) virulome within each group.

Genomes harboured a median of 5 AMR genes, with a minimum of 2 and a maximum of 17; 86.9% (*n*=100) of the genomes had between 5 and 6 AMR genes, and 14 genomes were above this AMR gene content value. *AxyXY-OprZ*, *ceoB* and subtypes of *bla*_OXA-114_ were identified as core-AMR genes, as they were detected in >95% of isolates (Fig. 5). Clinical isolates (all groups except environmental) showed a greater diversity of AMR genes, mainly *bla*_AXC-1_ β-lactamases and, to a lesser extent, *bla*_GES_, *bla*_IMP_, *bla*_NPS_, *bla*_OXA-10_ and aminoglycoside-modifying enzymes from the *aadA* and *APH* families, dihydrofolate reductase *dfrA17* and tetracycline efflux pump *tetG*. In contrast, the *bla*_AXC-4_ gene was unique to environmental isolates (Fig. 6a).

**Fig. 7.**
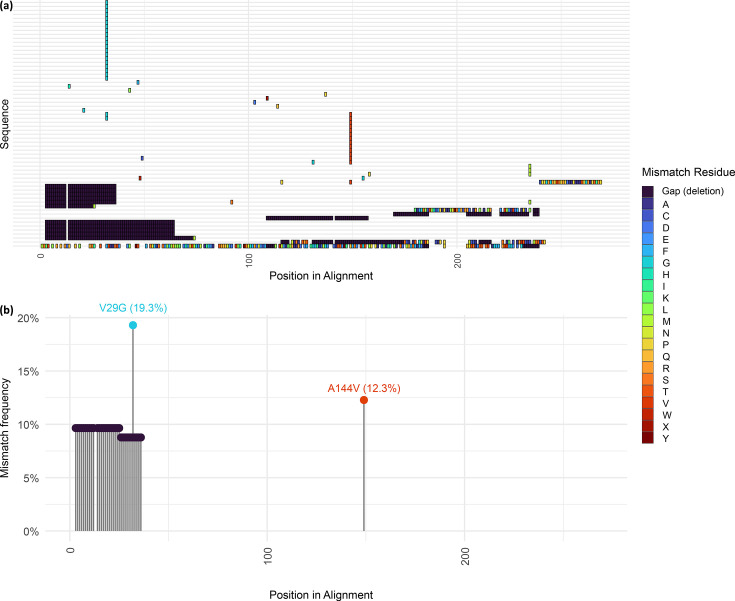
Variants found in the AxyZ protein sequences. (**a**) Distribution of mismatches across the length of the AxyZ sequence. Each row represents a genome of the analysed dataset. (**b**) Lollipop plot of the frequency of mutations (only frequencies greater than 5% are shown).

Detailed analysis of AxyXY-OprZ and its negative regulator AxyZ showed extensive variants in the structural components AxyXY-OprZ (Figs S3–S5). In contrast, three predominant variants were identified in the AxyZ negative regulator: V29G (19.3%), A144V (12.3%) and N-terminal deletions (9.6%) (Fig. 7). The V29G variant and N-terminal deletions in AxyZ were widely distributed among assemblies, with no preference for specific clones, but were limited to isolates of clinical origin (Fig. 5). The V29G variant is predicted to affect protein function according to the SIFT algorithm [49].

VF screening showed an average of 31 VF genes per genome. Most genomes harbour genes associated with the type VI secretion systems (*tssC*, *QU43_RS8250*) and flagella (*flgBCDFGHI*, *fliFIJ* and *flhABCD*). Genes related to the type III secretion system (*bcrD*, *bscSN* and *PA06860*) had variable frequencies across all isolation source groups. No association was observed between ST, isolation source or country of isolation and VF content. However, environmental and non-respiratory clinical isolates showed a higher frequency of LPS biosynthesis genes from the *wbmFGH locus* (Fig. 6b).

### Association between the mobilome and AMR genes

Mobilome-associated elements accounted for most accessory AMR genes in 13 of the 14 genomes that harboured more than six AMR genes. A significant association between mobilome elements and AMR gene content was detected (*P*=0.0035) (Table S2).

**Fig. 8.**
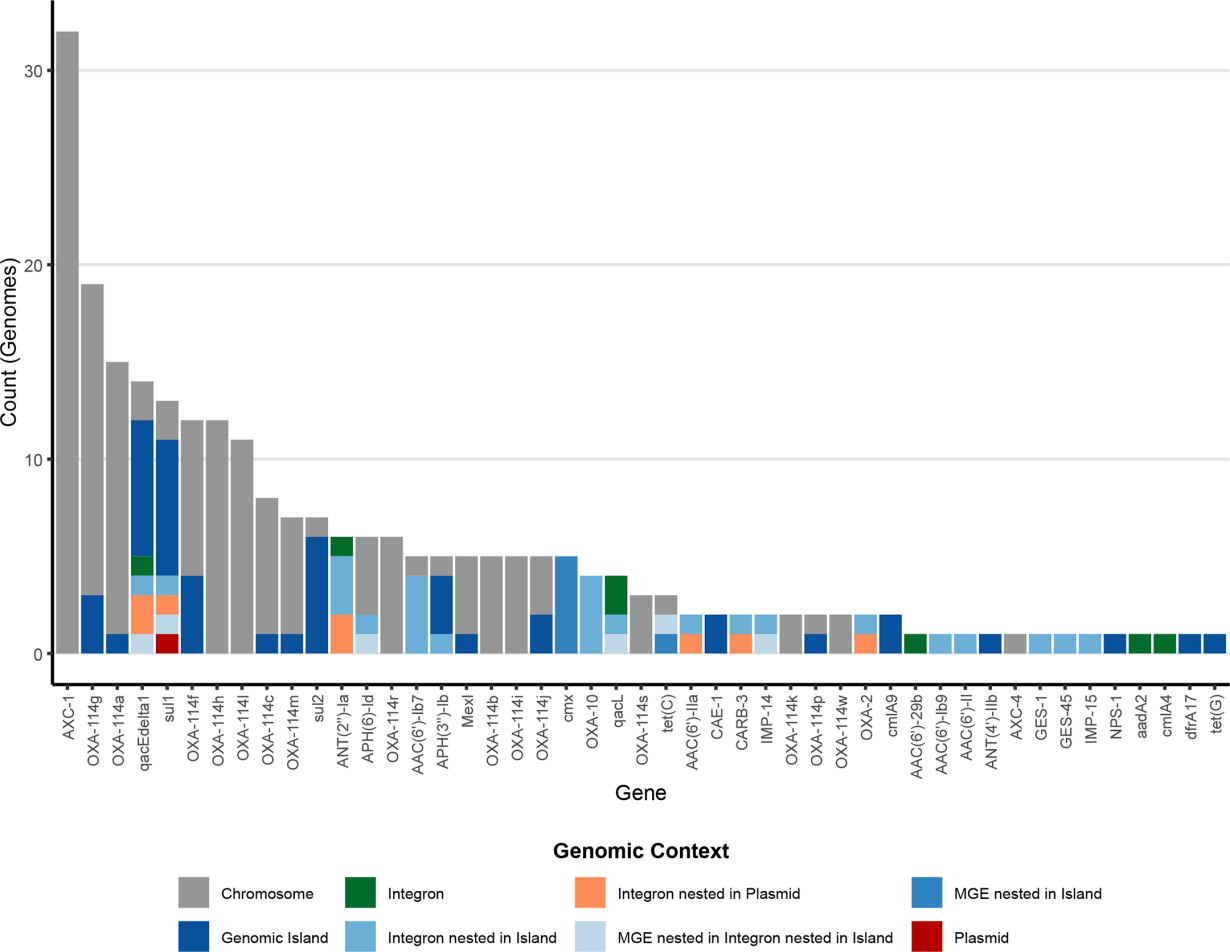
Distribution of AMR genes across mobilome elements. (**a**) Count of AMR genes according to their genetic origin. Core AMR genes (*AxyXY-OprZ*) are not shown for simplicity. (**b**) Alluvial plot showing the relationship between AMR genes and their genetic source.

To classify AMR genes according to their mobilome element of origin and accounting for the element nesting, resistome results derived from whole-genome assemblies were compared with those obtained from individually extracted mobilome elements. An AMR gene was considered to have chromosomal origin when it was absent from all analysed mobile elements of the corresponding genome, whereas genes detected in more than one mobilome element were classified as nested (Table 1). The final assignment of AMR elements according to their genetic source is shown in Fig. 8. A total of 27 GIs carried AMR genes in the analysed dataset, making GIs the mobilome element with the highest AMR burden, followed by integrons (*n*=13), MGEs (*n*=7) and plasmids (*n*=2).

**Table 1.** Frequency of AMR genes of mobilome origin

Mobilome element	AMR gene	Count
GI	*qacEdelta1*, *sul1*	7 (each)
	*sul2*	6
	*bla*_OXA-114f_	4
	*APH(3'')-Ib*, *bla*_OXA-114g_	3 (each)
	*bla*_CAE-1_, *bla*_OXA-114j_, *cmlA9*	2 (each)
	*ANT(4')-IIb*, *AxyX*, *bla_O_*_XA-114a_, *bla*_OXA-114c_, *bla*_OXA-114m_, *bla*_OXA-114p_, *bla*_NPS-1_ *dfrA17*, *tet(G)*, *MexI*	1 (each)
Integron	*qacL*	2
	*aadA2*, *AAC(6')−29b*, *ANT(2″)-Ia*, *ceoB*, *cmlA4*, *qacEdelta1*	1 (each)
Integron nested in GI	*AAC(6')-Ib7*, *bla*_OXA-10_	4 (each)
	*ANT(2″)-Ia*	3
	*AAC(6')-IIa*, *AAC(6')-Ib9*, *AAC(6')-Il*, *APH(3″)-Ib*, *APH(6)-Id*, *bla*_CARB-3_, *bla*_GES-1_, *bla*_GES-45_, *bla*_IMP-14_, *bla*_IMP-15_, *bla*_OXA-2_, *qacEdelta1*, *qacL, sul1*	1 (each)
Integron nested in plasmid	*ANT(2″)-Ia*, *qacEdelta1*	2 (each)
	*AAC(6')-Iia*, *bla*_OXA-2_, *bla*_CARB-3_, *sul1*	1 (each)
MGE nested in integron nested in GI	*APH(6)-Id*, *bla*_IMP-14_, *qacEdelta1*, *qacL*, *sul1*, *tet(C*)	1 (each)
MGE nested in GI	*cmx*	5
	*tet(C*)	1
Plasmid	*sul1*	1

The most frequently identified genes within GIs belonged to the *qac*, *sul1*, *sul2* and *bla*_OXA-114_ families. Notably, GIs commonly harboured additional mobile elements that carried AMR genes. Among these, integrons nested in GIs (*n*=9) carried genes from the *AAC*, *ANT*, *APH*, *bla*_GES_, *bla*_CARB_, *bla*_OXA_ and *bla*_IMP_ gene families.

A single occurrence of an MGE nested within an integron, which was itself embedded in a GI, was identified. This element carried *qacEdelta1*, *qacL*, *sul1*, *tet(C*) and *bla*_IMP-14_. MGEs nested in GIs were also identified (*n*=6), albeit at lower frequencies compared to integrons within GIs and were associated with *cmx* and *tet(C*).

Plasmids were uncommon, and few of them carried AMR genes (Fig. 8). Plasmids were detected in nine genomes, one of which carried two plasmid sequences. Four plasmid sequences harboured enzymes associated with mercury resistance, one of which also contained AMR genes [*bla*_CARB_, *AAC(6*′) and *aadB*] within an integron. Two other plasmids encoded proteins related to restriction system resistance, one of which also harboured *bla*_OXA-2_ within an integron. Another plasmid carried the VF genes *pld* and *pilT*, and another encoded enzymes for aromatic compound degradation. The remaining plasmid contained only hypothetical proteins with no assigned function. No relationship between the functional content, isolation source or ST was observed (Fig. S6).

Regarding phages, 720 sequences were detected among the analysed dataset, of which 345 had intact integrity, 50 were incomplete and 203 had questionable integrity. Clustering based on mash distance >0.4 revealed two major phage-like sequences that were widely distributed across isolates. The first sequence was identified by PHASTEST as *Salmon_SEN34_NC_28699*. The second cluster consisted of *Burkho_KS9_NC13055* and *Burkho_Bcep1766_NC7497*. Among all the sequences analysed, four phages contained VF genes, all associated with the flagellum. No AMR genes were detected within the phage sequences.

## Discussion

*A. xylosoxidans* has intrinsic resistance to a broad spectrum of antibiotics, through numerous resistance mechanisms [50]. In this study, we identified a limited and low-diversity resistome among publicly available *A. xylosoxidans* genomes, which was primarily composed of intrinsic *bla*_OXA-114_ subtypes, the AxyXY-OprZ efflux pump and, to a lesser extent, the *bla*_AXC-1_ carbapenemase, which was detected only in clinical isolates.

Overexpression of the AxyXY-OprZ efflux system has been associated with antibiotic resistance [7,9]; however, most studies have focused on documenting this overexpression without addressing the underlying regulating mechanisms [51]. In our dataset, we observed a high frequency of mutations of AxyZ, which is the negative regulator of the AxyXY-OprZ system. The most frequent variant in AxyZ was V29G, which is known to induce overexpression and reduce susceptibility to multiple antibiotics [9]. Furthermore, N-terminal deletions within the TetR regulatory domain of *AxyZ* were also detected. Given that this domain is needed for regulatory function, such deletions are likely to impair protein function and contribute to efflux-mediated resistance. Considering the polymorphic patterns observed among the AxyXY-OprZ components, the dominant AxyZ variants in clinical isolates might indicate that the attenuation of AxyZ-mediated negative regulation may trigger AMR.

Along with AxyXY-OprZ, *bla*_OXA-114_ subtypes were identified as core-AMR genes in *A. xylosoxidans*; these are narrow-spectrum, non-inducible class D *β*-lactamases that are characteristic of this species. *β*-Lactamases of the *bla*_OXA-114_ family remain largely understudied; reports indicate that they exhibit weak hydrolytic activity against most antibiotics and are susceptible to *β*-lactam inhibitors [52]. While attempts to associate specific *bla*_OXA-114_ subtypes with resistance patterns have been made, their role in *β*-lactam resistance is limited [53,54]. Besides its relevance as an AMR determinant, *bla*_OXA-114_ has been proposed as a species marker to differentiate *A. xylosoxidans* from other species of the genus, all of which code *bla*_OXA-114_ subtypes [55]. In this sense, 114 out of 115 of the included assemblies were positive for *bla*_OXA-114_, highlighting its ubiquity and supporting its role as a potential species marker.

Besides these previously mentioned core AMR genes, *bla*_AXC-1_ was the most frequently detected AMR gene in this dataset and was exclusively of chromosome origin. This *β*-lactamase exhibits mild carbapenemase activity [56,57] and was exclusively found in genomes of clinical origin, underscoring its potential clinical relevance.

Mobilome sequences were predominantly associated with AMR genes, particularly extended-spectrum *β*-lactamases such as *bla*_OXA-2_, *bla*_OXA-10_, *bla*_GES-1_, *bla*_GES-45_, *bla*_IMP-14_ and *bla*_IMP-15_. These determinants were exclusively mobilome-derived, predominantly from integrons, which is consistent with a large-scale analysis of 11,957 integron sequences, in which *bla*_OXA_, *bla*_GES_ and *bla*_IMP_ were identified among the most frequent AMR gene families [58]. Importantly, all mobilome-associated *β*-lactamases in our dataset were confined to clinical isolates and have also been reported in pathogens such as *Klebsiella pneumoniae* [59,60] and *Pseudomonas aeruginosa* [61,63], highlighting their potential clinical relevance.

AMR genes detected at lower frequency in our study included the aminoglycoside modification enzymes from the *AAC*, *ANT* and *APH* gene families. These genes have been reported to be widespread across diverse bacterial species and thought to be primarily exchanged between different biomes, particularly humans, clinical environments and agrosystems, rather than selected by aminoglycoside exposure [33,64]. Their dissemination is thought to rely on MGEs [64], as observed in our dataset. Aside from AMR genes, plasmids in both clinical and environmental isolates carried genes involved in resistance to aromatic compounds. However, due to the small sample size and low detection frequency of plasmids, no clear association between isolate characteristics and plasmid content can be drawn.

Although STs of *A. xylosoxidans* remain underreported, ST182 was the most frequent ST and has also been associated with *bla*_AXC-1_-positive strains in another study [57], as with our dataset. Along with ST182, most ST-defined clusters included isolates from different countries, suggesting the international dissemination of clones with conserved core genomes. The overall recombination-masked SNP distance across the collection was 32,013 SNPs, substantially higher than the average intra-ST cluster distance of 142 SNPs. This sharp contrast, together with the close relatedness observed in the core-genome phylogeny, supports the emergence of clones within *A. xylosoxidans*.

Numerous pathways were uniquely enriched within the accessory genome of isolates according to their isolation source. Non-CF respiratory isolates were enriched in pathways related to secretion systems and fatty acid metabolism. This goes in line with the results of Le Goff *et al*., in which a respiratory isolate of *A. xylosoxidans* had higher expression of type VI secretion system effectors compared to a bloodstream isolate [65]. In addition, genes involved in iron acquisition, particularly regulators and the ferripyoverdine receptor, were unique to the non-CF respiratory isolates. Iron is essential for microbial growth, and host–pathogen competition for this metal is well documented at mucosal surfaces, such as the airway epithelium [63]. These findings support a potential role of iron acquisition systems in adaptation to the respiratory niche for *A. xylosoxidans*. Accordingly, clinical isolates of *A. xylosoxidans* have been reported to harbour and produce higher levels of siderophores than environmental isolates [66]. Clinical non-respiratory isolates were enriched in drug metabolism and chemotaxis pathways. Chemotaxis plays an important role in establishing infections, allowing bacteria to migrate toward nutrient-rich environments, respond to host gradients and invade tissues [67]. On the other hand, CF isolates were enriched in nucleotide sugar biosynthesis, which are essential for bacterial cell wall and envelope formation, maintaining structure and providing protection against the environment and host-derived stress [68]. This finding contrasts with a genomic study of CF isolates, which found that chronic strains lack genes involved in O-antigen and LPS synthesis [69]. This discrepancy may be due to our exclusion of genomes from longitudinal studies. In our study, we excluded longitudinal isolates obtained from public databases, which likely include repeated isolates from the same patients, to minimize sampling bias. Their overrepresentation would have introduced redundancy into the dataset, and de-duplication was not possible due to the absence of patient identifiers in the public databases.

Regarding the genomic characteristics, *A. xylosoxidans* exhibits an average genome size of ~6.6 Mbp, with a content of 5,948 genes, of which 4,275 (~71%) were part of the core genome. In comparison, other environmentally adaptable opportunistic pathogens, such as *P. aeruginosa*, have a similar genome size (~6.6 Mbp) but a smaller core genome (~3,796 genes) [70]. On the other hand, species recognized as ‘specialists’ due to their restricted ecological niches, such as *Bordetella pertussis*, a strict human pathogen, possess a reduced genome (~4.0 Mbp) with ~3,600 core genes [71]. Similarly, *Staphylococcus aureus* has a more compact genome (~2.8 Mbp) and a smaller core genome (~2,000 genes) [72].

Larger genome sizes and expanded core genomes have been associated with ecological ubiquity and enhanced capacity to persist and thrive across diverse environments [73,74]. This is also supported by the enrichment of numerous metabolic and biosynthetic pathways within the accessory genome [75]. Furthermore, while the biological implications of GC content remain under debate, GC-rich genomes have been associated with increased recombination rate and, thus, genomic plasticity [76,77].

Among the most frequently detected prophages in this study, Salmon_SEN34 has been linked to multidrug-resistant *Salmonella enterica*. In contrast, Burkho_KS9 and Burkho_Bcep176 have been reported as frequent in CF isolates but lack known virulence or resistance functions [11,78]. Burkho_BcepMu, though linked to potential type II secretion system toxins [79], did not encode these factors in our dataset. Beyond gene cargo, prophages can influence bacterial fitness by conferring protection against exogenous phages and shaping competitive interactions within microbial communities [80]. This is supported by the fact that these prophages are annotated as lytic by the UniProt database.

No VFs associated with MGEs were detected. In contrast, a prior study reported VF-carrying elements in *Achromobacter* spp. [11]. This discrepancy likely stems from differences in the annotation strategies used. Veschetti *et al*. used broad protein-family level annotations, which often include general functional categories, whereas our approach utilized the VFDB dataset, prioritizing specificity for VF identification, at the expense of sensitivity.

Beyond their role as gene vectors for gene transfer, MGEs may also influence gene expression and genome plasticity through insertional mutagenesis or regulatory disruption [81]. An example in our dataset was the IS ISButh3, which does not encode AMR or VF genes yet was present in 24 copies within isolate GCF_032703345. Although this finding suggests a potential impact on genome architecture, a detailed analysis of insertional mutagenesis was beyond the scope of this study.

This study has limitations. First, KEGG pathway enrichment results should be interpreted with caution, as they reflect gene presence rather than actual gene expression or functional activity. Second, our phage identification relied primarily on similarity-based approaches, which may underestimate the diversity of novel or highly divergent elements. Third, GI prediction is prone to false positives, particularly when multiple mobilome elements co-occur within a genome, and thus should be interpreted with caution. Fourth, the absence of phenotypic data limits our ability to link genomic characteristics with phenotypes, and finally, we discarded longitudinal follow-up isolates of the same strain, which may have led to the loss of information regarding the acquisition, retention, loss or selection of environmental genes across time.

In conclusion, we provide a comprehensive genomic overview of the resistome, mobilome and functional diversification of * A. xylosoxidans*. This species harbours a relatively small resistome; we suggest that AMR is likely driven in by disruption of the AxyZ negative regulator in clinical settings, leading to an increased expression of efflux pumps. Mobilome-associated AMR genes were uncommon and only detected in clinical isolates. The combination of a large, plastic genome with broad metabolic versatility and potential to acquire AMR genes through HGT underscores the potential of this species to thrive across environments and evade antimicrobial interventions.

## Supplementary material

10.1099/mgen.0.001744 Supplementary Material 1.

## Abbreviations

AMR: antimicrobial resistance
ANI: average nucleotide identity
CDS: coding sequence
CF: cystic fibrosis
COG: Clusters of Orthologous Genes
GI: genomic island
HGT: horizontal gene transfer
IS: insertion sequence
MGE: mobile genetic element
ML: maximum-likelihood
MLST: multilocus sequence typing
ST: sequence type
VF: virulence factor
VFDB: Virulence Factor Database
