# Supplementary material for: Genomic insights into the resistome, mobilome and functional adaptation of Achromobacter xylosoxidans across clinical and environmental contexts
Source: Microb Genom. 2026 Jun 29;12(6):001744. doi: 10.1099/mgen.0.001744 (PMC13313594; doi:10.1099/mgen.0.001744)
Supplement: Supplementary Material 1. [file mgen-12-01744-s001.pdf]

Supplementary Figure 1. Average Nucleotide Identity of the included assemblies and type strains of *Achromobacter* species.

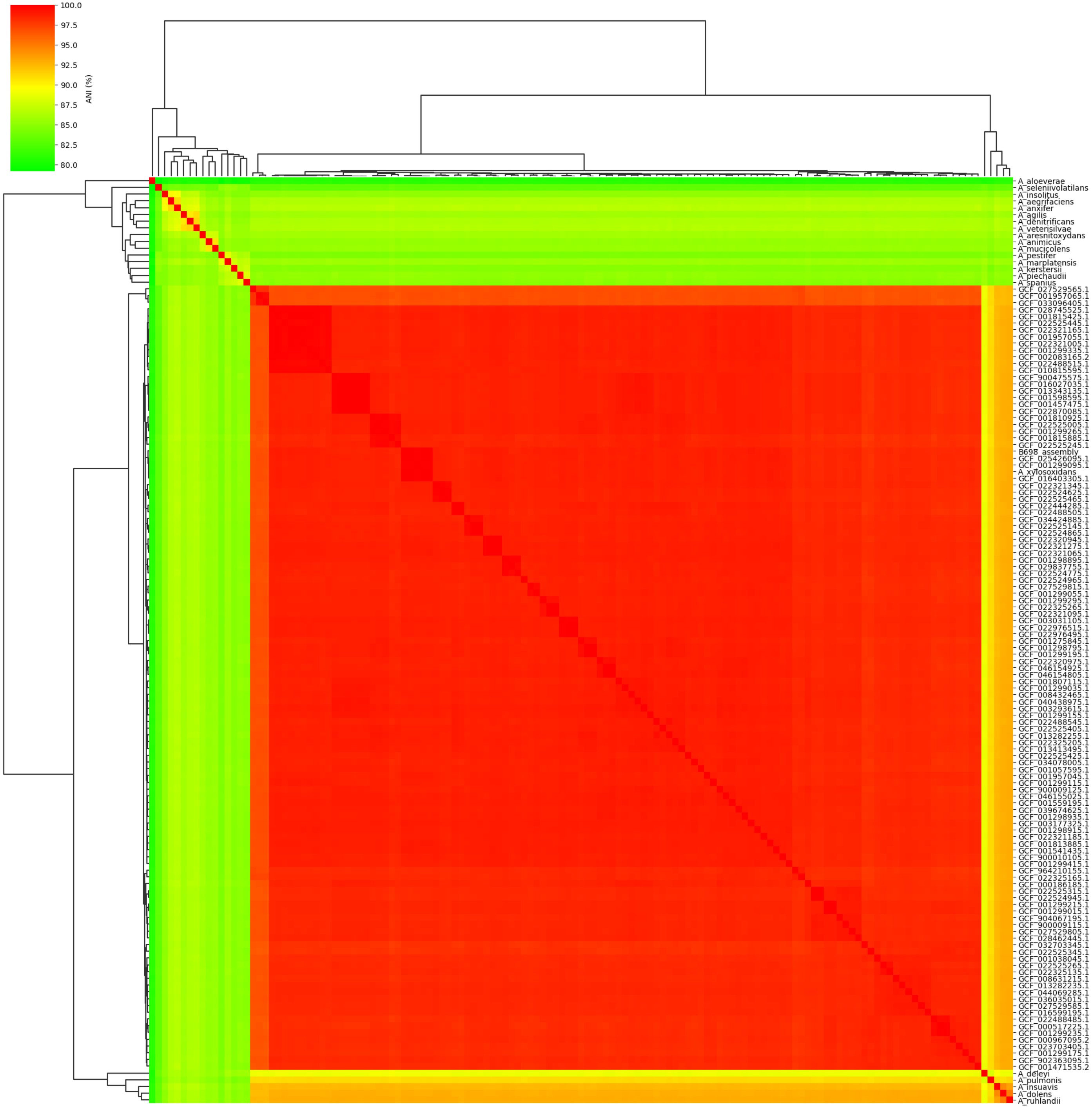

Supplementary Figure 2. Average Nucleotide Identity of assemblies with inconclusive taxonomy and type strains of *Achromobacter* species.

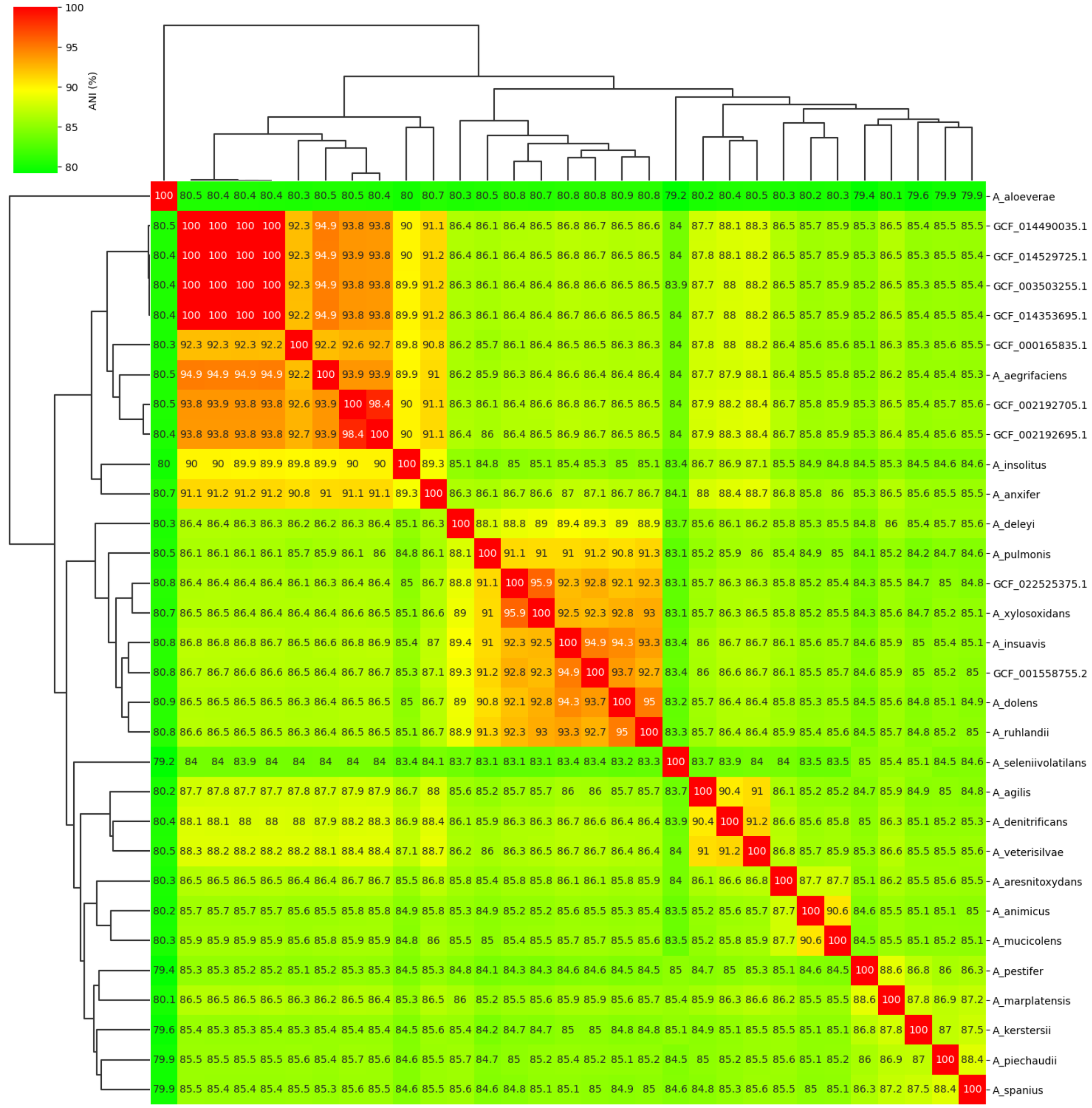

Supplementary Figure 3. Lollipop plot of AxyX variants.

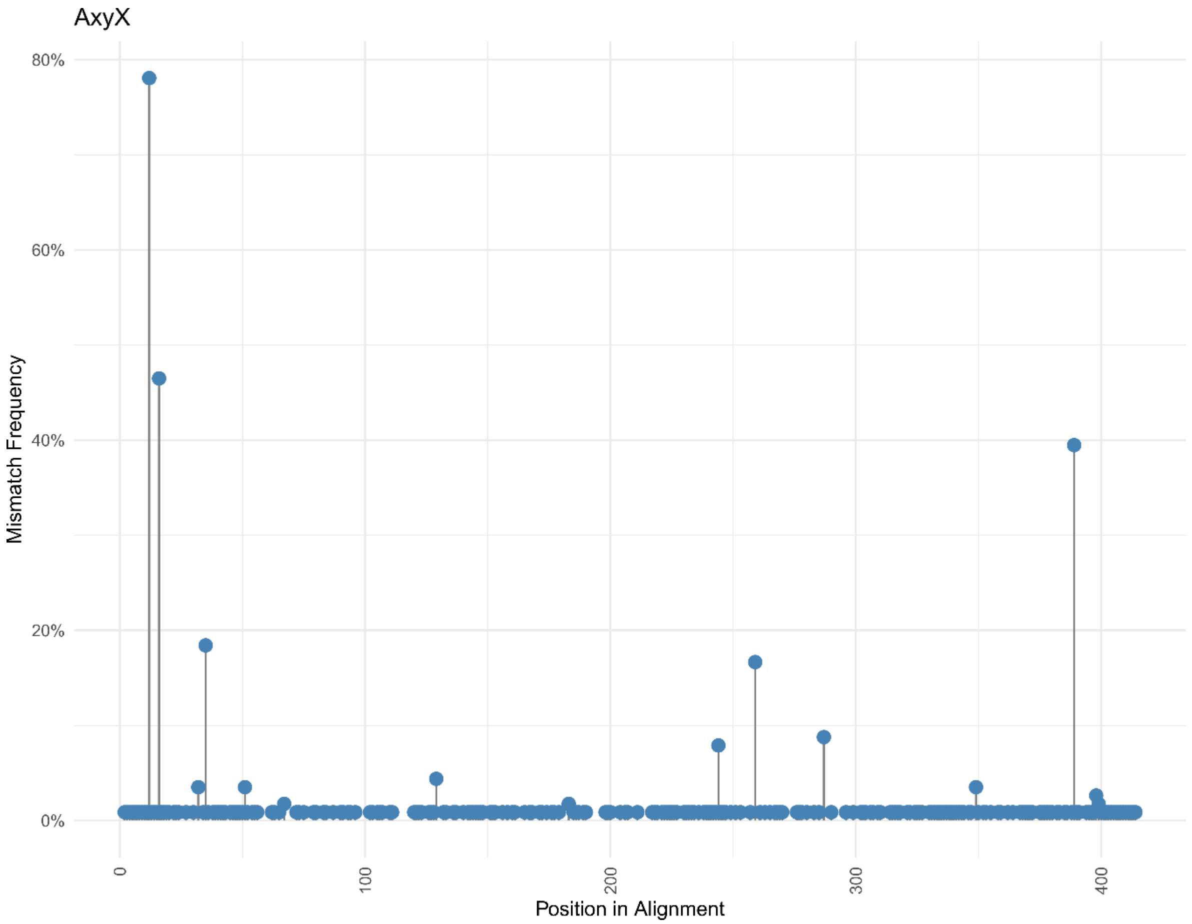

Supplementary Figure 4. Lollipop plot of AxyY variants.

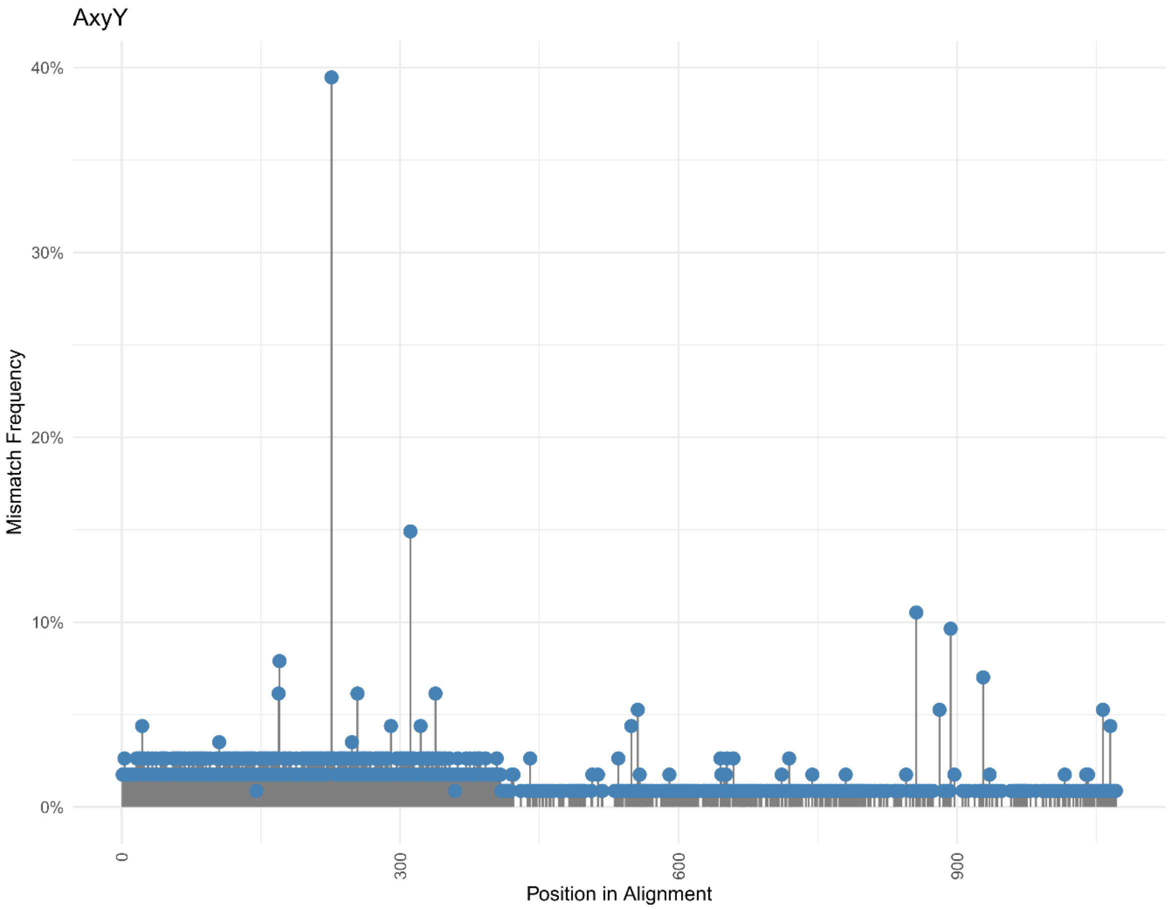

Supplementary Figure 5. Lollipop plot of OprZ variants.

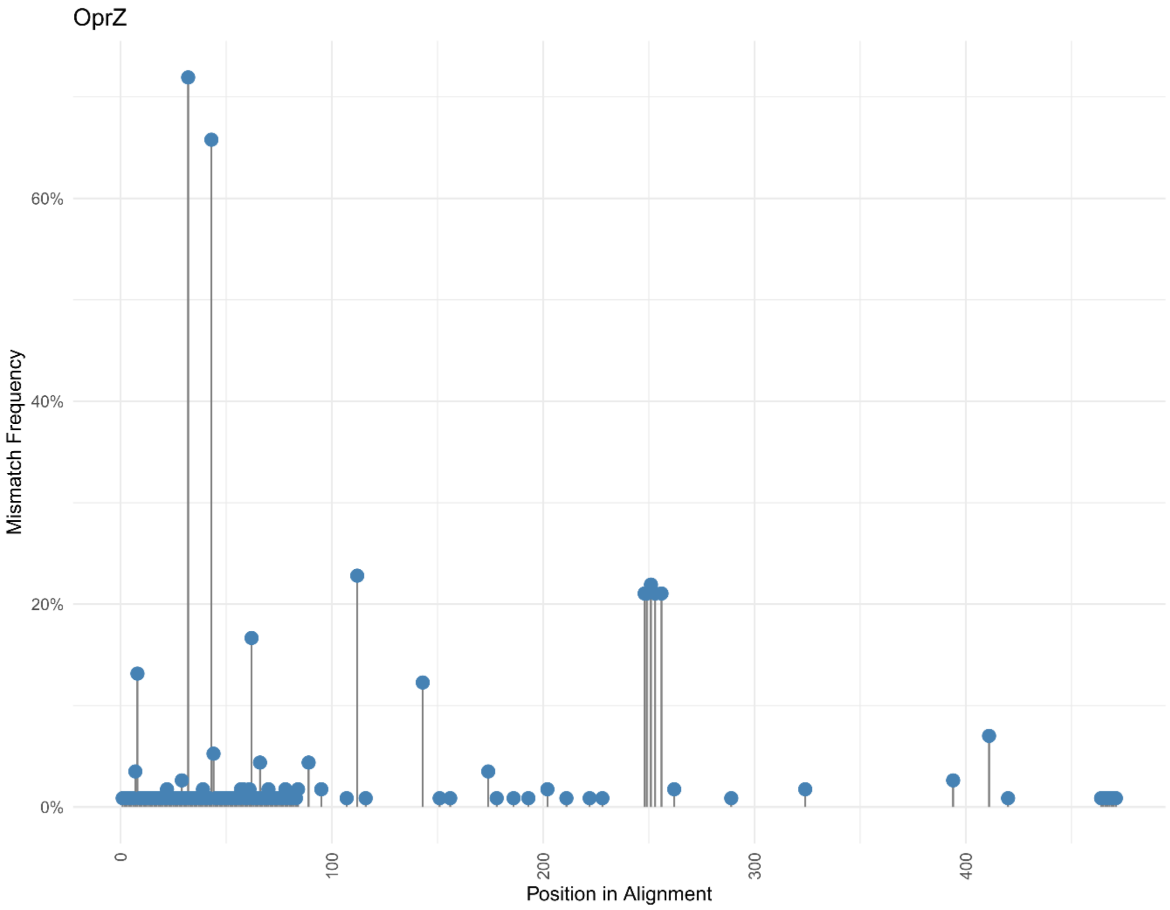

Supplementary Figure 6. Plasmid type distribution. Plasmids were classified according to the genes they contained.

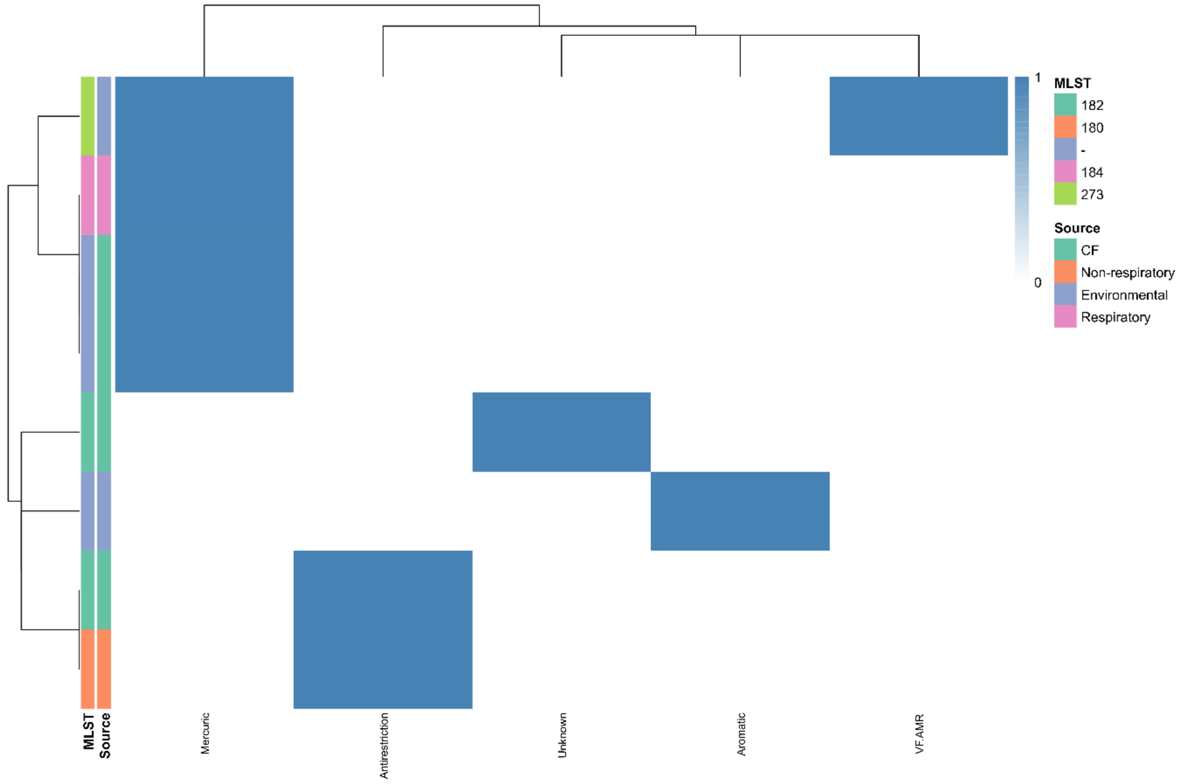

Supplementary Table 1. Metadata of the included assemblies from NCBI RefSeq.

Genome	Country	Date	Source	MLST	Bioproject	Completeness	Contamination	# Contigs	N50	L50
B698	Mexico	Missing	CF	290	PRJNA1301411	99.53	0.58	47	502802	5
GCF_000186185.1	Missing	Missing	Respiratory	Novel	PRJNA38739	94.07	1.4	262	137710	10
GCF_000517225.1	China	2010	Respiratory	314	PRJNA209574	99.07	0.58	116	123213	18
GCF_000967095.2	Missing	Missing	CF	Novel	PRJNA40627	99.53	0.93	1	6916670	1
GCF_001038045.1	Canada	2011	CF	Novel	PRJNA287154	99.53	0.47	29	586493	3
GCF_001057595.1	USA	Missing	CF	Novel	PRJNA267549	99.53	0.99	725	16540	109
GCF_001275845.1	Australia	2006	CF	Novel	PRJNA285410	99.47	0.47	514	21396	88
GCF_001298795.1	USA	2013	CF	Novel	PRJEB4659	98.12	0	33	465724	4
GCF_001298895.1	USA	2013	CF	2	PRJEB4659	98.86	0.47	76	229357	8
GCF_001298915.1	USA	2013	CF	Novel	PRJEB4659	99.07	0.47	21	543642	4
GCF_001298935.1	USA	2013	CF	Novel	PRJEB4659	98.68	0.47	27	468071	5
GCF_001299015.1	USA	2013	CF	Novel	PRJEB4659	98.68	0.47	37	415223	5
GCF_001299035.1	USA	2013	CF	Novel	PRJEB4659	98.68	0.58	50	338662	6
GCF_001299055.1	USA	2013	CF	426	PRJEB4659	98.83	0.47	56	292081	8
GCF_001299095.1	USA	2013	CF	290	PRJEB4659	98.71	0.47	35	475428	5
GCF_001299115.1	USA	2013	Non-respiratory	Novel	PRJEB4659	99.3	0.47	28	417570	5
GCF_001299155.1	USA	2013	CF	Novel	PRJEB4659	98.91	0.47	28	937183	3
GCF_001299175.1	USA	2013	CF	Novel	PRJEB4659	99.14	0.47	47	412048	6
GCF_001299195.1	USA	2013	CF	Novel	PRJEB4659	99.38	0	106	194741	10
GCF_001299215.1	USA	2013	CF	Novel	PRJEB4659	99.38	0.47	30	858782	3
GCF_001299235.1	USA	2013	CF	314	PRJEB4659	98.87	0.47	28	867581	3
GCF_001299265.1	USA	2013	CF	175	PRJEB4659	98.61	0.47	45	348171	7
GCF_001299295.1	USA	2013	CF	Novel	PRJEB4659	99.3	0.47	30	760952	4
GCF_001299335.1	USA	2013	CF	182	PRJEB4659	97.27	0.93	35	381188	6
GCF_001299415.1	USA	2013	CF	Novel	PRJEB4659	98.6	0.93	21	770680	4
GCF_001457475.1	Japan	1971	Non-respiratory	20	PRJEB6403	99.53	0.58	1	6813182	1
GCF_001471535.2	USA	2014	CF	Novel	PRJNA231221	99.53	2.73	8	6233405	1
GCF_001541435.1	Mexico	2013	Environmental	Novel	PRJNA301638	99.53	0.47	26	1159672	3
GCF_001559195.1	US	2014	CF	Novel	PRJNA231221	99.53	0.47	1	6268754	1
GCF_001598595.1	Missing	Missing	Non-respiratory	20	PRJDB238	99.53	0.58	63	201844	11
GCF_001807115.1	Missing	Missing	Non-respiratory	Novel	PRJNA269874	99.26	0.53	356	37050	50
GCF_001810925.1	Missing	Missing	Non-respiratory	175	PRJNA299926	99.53	0.47	77	255847	10
GCF_001813885.1	Missing	Missing	Respiratory	Novel	PRJNA300093	98.6	0.47	59	404527	5
GCF_001815425.1	Missing	Missing	Respiratory	182	PRJNA300177	98.88	0.47	71	314966	6
GCF_001815885.1	Missing	Missing	Respiratory	175	PRJNA269806	99.29	0.53	1012	12588	142
GCF_001957045.1	Australia	2006	CF	Novel	PRJNA343957	99.07	0.7	61	275436	7
GCF_001957055.1	Canada	2014	Respiratory	182	PRJNA343957	99.53	0.47	42	358534	6
GCF_001957065.1	Australia	2005	Respiratory	Novel	PRJNA343957	99.53	0.93	65	209421	8
GCF_002083165.2	USA	2014	Non-respiratory	182	PRJNA231221	99.53	0.93	1	6743846	1

GCF_003031105.1	China	Missing	Environmental	327	PRJNA362984	99.53	0.47	29	734314	3
GCF_003177325.1	Brazil	2005	CF	Novel	PRJNA453449	99.53	0.58	128	148162	15
GCF_003293615.1	Brazil	2016	Environmental	Novel	PRJNA391768	99.53	0.47	2	6300041	1
GCF_008432465.1	China	2018	Respiratory	Novel	PRJNA563576	99.53	0.47	1	6402982	1
GCF_008631215.1	Oman	2015	Environmental	Novel	PRJNA564585	99.53	0.93	15	1032880	2
GCF_010815595.1	USA	2019	Non-respiratory	182	PRJNA591881	99.53	0.47	335	38162	53
GCF_013282235.1	USA	2010	Non-respiratory	Novel	PRJNA604252	99.53	0.47	1	6452474	1
GCF_013282255.1	USA	2010	CF	418	PRJNA604252	99.53	0.47	1	6608203	1
GCF_013343135.1	Germany	Missing	Missing	20	PRJNA231221	99.53	0.58	1	6806438	1
GCF_013413495.1	China	Missing	Environmental	Novel	PRJNA644672	99.53	0.58	44	611861	3
GCF_016027035.1	Missing	Missing	Missing	20	PRJNA231221	99.38	0.58	1	6812921	1
GCF_016403305.1	Germany	Missing	Missing	290	PRJNA231221	99.53	0.47	1	6894935	1
GCF_016599195.1	India	2016	Non-respiratory	Novel	PRJNA686214	99.53	0.47	2	6528153	1
GCF_022320945.1	Russia	2020	CF	346	PRJNA799738	99.53	0.47	33	415563	5
GCF_022320975.1	Russia	2020	CF	Novel	PRJNA799738	99.53	0.47	29	1206806	3
GCF_022321005.1	Russia	2020	CF	182	PRJNA799738	99.53	0.47	46	422057	5
GCF_022321065.1	Russia	2020	CF	346	PRJNA799738	99.53	0.47	35	561073	5
GCF_022321095.1	Russia	2020	CF	530	PRJNA799738	99.3	0.47	68	253029	8
GCF_022321165.1	Russia	2020	CF	182	PRJNA799738	99.53	0.47	37	468297	4
GCF_022321185.1	Russia	2020	CF	Novel	PRJNA799738	99.53	0.58	35	554162	4
GCF_022321275.1	Russia	2020	CF	346	PRJNA799738	99.53	0.47	37	610824	4
GCF_022321345.1	Russia	2020	CF	236	PRJNA799738	99.53	0.47	37	406130	5
GCF_022325135.1	Russia	2020	CF	Novel	PRJNA799738	99.53	0.47	41	436835	5
GCF_022325165.1	Russia	2020	CF	Novel	PRJNA799738	99.53	0.47	35	605514	4
GCF_022325205.1	Russia	2020	CF	Novel	PRJNA799738	99.53	0.47	25	718711	3
GCF_022325265.1	Russia	2020	CF	530	PRJNA799738	99.3	0.47	36	503962	4
GCF_022444285.1	Serbia	2012	CF	417	PRJNA801228	99.53	1.61	163	90105	23
GCF_022488485.1	Serbia	2012	CF	314	PRJNA801228	99.53	14.67	603	74647	33
GCF_022488505.1	Serbia	2012	CF	417	PRJNA801228	99.53	0.88	460	27327	74
GCF_022488515.1	Serbia	2012	CF	182	PRJNA801228	99.53	0.58	354	31670	65
GCF_022488545.1	Serbia	2012	CF	418	PRJNA801228	100	1.23	150	82282	25
GCF_022524625.1	Germany	2017	CF	Novel	PRJNA782431	99.42	0.93	666	16572	116
GCF_022524775.1	Germany	2016	CF	2	PRJNA782431	99.53	0.93	267	47629	43
GCF_022524865.1	Germany	2015	CF	184	PRJNA782431	99.53	0.93	221	56734	37
GCF_022524945.1	Germany	2015	CF	Novel	PRJNA782431	99.53	0.93	670	17602	121
GCF_022524965.1	Germany	2014	CF	Novel	PRJNA782431	99.53	0.58	491	23877	92
GCF_022525005.1	Germany	2014	CF	175	PRJNA782431	99.53	1.46	320	37227	54
GCF_022525145.1	Germany	2011	CF	Novel	PRJNA782431	99.53	0.53	210	61124	34
GCF_022525245.1	Germany	2010	CF	Novel	PRJNA782431	99.38	1.23	972	11747	175
GCF_022525265.1	Germany	2010	CF	Novel	PRJNA782431	99.53	0.93	283	45838	45
GCF_022525315.1	Germany	2010	CF	Novel	PRJNA782431	99.07	0.93	555	20963	104

GCF_022525345.1	Germany	2009	CF	Novel	PRJNA782431	99.07	0.53	615	19510	108
GCF_022525405.1	Germany	2008	CF	Novel	PRJNA782431	99.07	0.99	563	19404	101
GCF_022525425.1	Germany	2007	CF	Novel	PRJNA782431	99.53	0.93	333	33736	56
GCF_022525445.1	Germany	2006	CF	182	PRJNA782431	99.53	0.47	284	39106	50
GCF_022525465.1	Germany	2009	CF	236	PRJNA782431	99.07	0.53	759	15302	127
GCF_022870085.1	Missing	Missing	Non-respiratory	20	PRJDB238	99.38	0.58	1	6813185	1
GCF_022976495.1	France	2012	CF	327	PRJNA823997	99.53	0.47	86	144745	16
GCF_022976515.1	France	2012	CF	327	PRJNA823997	99.53	0.47	120	98915	23
GCF_023703405.1	Argentina	2017	Non-respiratory	Novel	PRJNA843093	99.53	0.58	129	129032	18
GCF_025426095.1	USA	2021	Non-respiratory	290	PRJNA812595	99.53	0.47	1	6611782	1
GCF_027529565.1	Russia	2022	CF	Novel	PRJNA561493	99.53	0.47	76	174221	12
GCF_027529585.1	Russia	2022	CF	Novel	PRJNA561493	99.53	0.47	73	194803	11
GCF_027529805.1	Russia	2022	CF	Novel	PRJNA561493	99.53	0.47	71	196637	12
GCF_027529815.1	Russia	2022	CF	426	PRJNA561493	99.53	0.7	117	164902	11
GCF_028462445.1	Russia	2022	CF	Novel	PRJNA561493	99.53	0.47	54	239560	10
GCF_028745525.1	Russia	2019	CF	182	PRJNA561493	99.53	0.47	303	38432	51
GCF_029837755.1	USA	2018	Environmental	2	PRJNA868296	98.75	0.47	106	145874	16
GCF_032703345.1	China	2020	Environmental	Novel	PRJNA940286	99.53	0.47	1	6939500	1
GCF_033096405.1	Japan	2022	Environmental	Novel	PRJDB15726	99.53	0.93	1	7071873	1
GCF_034078005.1	Nigeria	2022	Environmental	Novel	PRJNA1043399	99.07	0.47	1	6930067	1
GCF_034424885.1	Argentina	2023	Respiratory	184	PRJNA1051020	99.53	0.58	216	64505	29
GCF_036035015.1	USA	2021	Non-respiratory	Novel	PRJNA1065584	99.53	0.47	31	404708	5
GCF_039674625.1	Australia	2021	Environmental	Novel	PRJNA964453	99.53	0.47	74	228162	11
GCF_040438975.1	China	2022	Environmental	Novel	PRJNA1097799	99.53	0.93	1	6650369	1
GCF_044069285.1	Germany	2023	Non-respiratory	Novel	PRJNA867390	99.53	1.21	45	529109	4
GCF_046154805.1	Russia	2023	CF	Novel	PRJNA561493	99.53	0.47	67	233963	11
GCF_046154925.1	Russia	20223	CF	Novel	PRJNA561493	99.53	0.47	98	236433	11
GCF_046155025.1	Russia	2023	CF	Novel	PRJNA561493	99.53	1.87	66	279012	7
GCF_900009115.1	Missing	Missing	Environmental	Novel	PRJEB11308	99.53	0.47	1	6501194	1
GCF_900009125.1	Thailand	2012	Non-respiratory	Novel	PRJEB11387	99.53	1.4	1	6626924	1
GCF_900010105.1	Thailand	2010	Non-respiratory	Novel	PRJEB11392	99.53	1.4	1	6690584	1
GCF_900475575.1	Missing	Missing	Non-respiratory	Novel	PRJEB6403	99.53	0.58	1	6735421	1
GCF_902363095.1	United Kingdom	Missing	Non-respiratory	Novel	PRJEB33885	99.53	0.47	18	710199	4
GCF_904067195.1	France	2017	Respiratory	Novel	PRJEB39103	99.53	0.78	37	392476	6
GCF_964210155.1	Tanzania	2021	Non-respiratory	Novel	PRJEB78444	99.53	0.47	370	32781	59

Supplementary Table 2. Total non-antimicrobial resistance genes and AMR gene count per genome, classified by genetic origin as mobilome-associated or chromosomal.

Genome	Chromosome origin		Mobilome origin	
	Non-AMR genes	AMR genes	Non-AMR genes	AMR genes
B698	5,681	7	490	8
GCF_000186185.1	5,592	5	345	0
GCF_000517225.1	5,830	6	713	5
GCF_000967095.2	6,346	6	0	0
GCF_001038045.1	5,427	5	288	0
GCF_001057595.1	5,602	2	453	0
GCF_001275845.1	5,462	6	478	0
GCF_001298795.1	5,651	6	629	0
GCF_001298895.1	5,500	5	364	0
GCF_001298915.1	5,390	6	333	0
GCF_001298935.1	5,527	5	333	0
GCF_001299015.1	5,455	5	252	0
GCF_001299035.1	5,506	6	526	1
GCF_001299055.1	5,918	6	498	0
GCF_001299095.1	5,737	5	393	0
GCF_001299115.1	5,505	5	324	0
GCF_001299155.1	5,486	5	379	0
GCF_001299175.1	5,576	6	468	0
GCF_001299195.1	5,591	6	689	0
GCF_001299215.1	5,487	5	217	0
GCF_001299235.1	5,684	6	456	0
GCF_001299265.1	5,742	5	508	0
GCF_001299295.1	5,563	6	448	0
GCF_001299335.1	5,683	6	594	0
GCF_001299415.1	5,473	5	304	0
GCF_001457475.1	6,127	6	0	0
GCF_001471535.2	5,763	9	366	0
GCF_001541435.1	5,522	5	337	0
GCF_001559195.1	5,655	5	0	0
GCF_001598595.1	5,595	5	535	1
GCF_001807115.1	5,564	5	458	0
GCF_001810925.1	5,676	5	452	0
GCF_001813885.1	5,466	5	341	0
GCF_001815425.1	5,488	5	409	0
GCF_001815885.1	5,521	5	805	0
GCF_001957045.1	5,486	5	351	0

GCF_001957055.1	5,718	6	521	0
GCF_001957065.1	5,856	5	680	0
GCF_002083165.2	6,119	6	0	0
GCF_003031105.1	5,591	5	226	0
GCF_003177325.1	5,421	5	270	0
GCF_003293615.1	5,354	6	476	0
GCF_008432465.1	5,699	5	0	0
GCF_008631215.1	5,591	5	272	0
GCF_010815595.1	5,433	6	495	0
GCF_013282235.1	5,780	5	0	0
GCF_013282255.1	5,973	6	0	0
GCF_013343135.1	6,119	6	0	0
GCF_013413495.1	5,562	5	449	6
GCF_016027035.1	6,125	6	0	0
GCF_016403305.1	6,279	5	0	0
GCF_016599195.1	5,436	5	434	0
GCF_022320945.1	5,501	5	472	0
GCF_022320975.1	5,588	5	659	0
GCF_022321005.1	5,718	6	593	0
GCF_022321065.1	5,500	5	339	0
GCF_022321095.1	5,912	6	0	0
GCF_022321165.1	5,572	6	483	0
GCF_022321185.1	5,573	7	251	3
GCF_022321275.1	5,478	5	296	0
GCF_022321345.1	5,537	5	596	0
GCF_022325135.1	5,325	5	339	0
GCF_022325165.1	5,548	5	172	0
GCF_022325205.1	5,514	5	413	0
GCF_022325265.1	5,905	6	0	0
GCF_022444285.1	5,276	6	1,027	4
GCF_022488485.1	4,846	6	1,557	0
GCF_022488505.1	5,500	5	742	5
GCF_022488515.1	5,782	6	676	5
GCF_022488545.1	5,450	6	610	5
GCF_022524625.1	5,846	5	2	0
GCF_022524775.1	5,553	5	605	0
GCF_022524865.1	5,584	6	376	0
GCF_022524945.1	5,280	5	688	0
GCF_022524965.1	5,930	9	6	5
GCF_022525005.1	6,141	5	7	0
GCF_022525145.1	5,959	6	0	0

GCF_022525245.1	6,026	5	9	0
GCF_022525265.1	6,135	5	3	0
GCF_022525315.1	5,327	5	669	0
GCF_022525345.1	5,677	6	529	0
GCF_022525405.1	5,306	5	394	0
GCF_022525425.1	5,507	5	399	0
GCF_022525445.1	5,075	6	592	0
GCF_022525465.1	5,404	5	549	0
GCF_022870085.1	6,113	6	11	0
GCF_022976495.1	5,597	6	284	0
GCF_022976515.1	5,580	6	299	0
GCF_023703405.1	5,764	7	481	5
GCF_025426095.1	5,992	5	0	0
GCF_027529565.1	5,393	5	311	0
GCF_027529585.1	5,497	5	266	0
GCF_027529805.1	5,442	5	524	0
GCF_027529815.1	5,664	6	594	0
GCF_028462445.1	5,351	5	408	0
GCF_028745525.1	5,467	6	401	0
GCF_029837755.1	6,522	5	15	0
GCF_032703345.1	6,335	5	0	0
GCF_033096405.1	6,423	5	0	0
GCF_034078005.1	6,365	5	0	0
GCF_034424885.1	5,630	7	543	6
GCF_036035015.1	5,503	5	268	0
GCF_039674625.1	5,511	5	225	0
GCF_040438975.1	5,996	5	0	0
GCF_044069285.1	5,421	5	422	0
GCF_046154805.1	5,415	5	278	0
GCF_046154925.1	5,560	5	479	0
GCF_046155025.1	5,619	5	342	0
GCF_900009115.1	5,861	5	0	0
GCF_900009125.1	5,970	12	0	0
GCF_900010105.1	6,087	17	0	0
GCF_900475575.1	6,064	6	0	0
GCF_902363095.1	5,586	6	528	0
GCF_904067195.1	5,364	5	516	0
GCF_964210155.1	5,452	5	319	1
Total	651,152	646	39,926	60
